# Effect of skill-based educational training for ambulance personnel on neonatal transport for newborn care in coastal South India – a single arm intervention study

**DOI:** 10.12688/f1000research.150058.1

**Published:** 2024-07-08

**Authors:** Santosh Kalyan, Sowmini Padmanabh Kamath, Subhodh Shetty S, Ramesh Holla, Leslie Lewis, Harsha Lashkari P, Suchitra Shenoy M, Shantharam Baliga B

**Affiliations:** 1 Department of Pediatrics, Kasturba Medical College, Mangalore, Manipal Academy of Higher Education, Karnataka, Manipal, 576104, India; 2 Department of Neonatology, Kasturba Medical College, Manipal Academy of Higher Education, Karnataka, Manipal, 576104, India; 3 Department of Community Medicine, Kasturba Medical College, Mangalore, Manipal Academy of Higher Education, Karnataka, Manipal, 576104, India; 4 Department of Pediatrics, Kasturba Medical College, Manipal Academy of Higher Education, Karnataka, Manipal, 576104, India; 5 Department of Microbiology, Kasturba Medical College, Mangalore, Manipal Academy of Higher Education, Karnataka, Manipal, 576104, India

**Keywords:** Ambulances, Checklist, Hypothermia, Hypoglycemia, Infant, Intensive care units, Neonatal, Newborn

## Abstract

**Background:**

Education of ‘108' ambulance personnel involved in transporting neonates may improve outcomes. We assessed i) perceptions/practices of ‘108’ ambulance personnel for transporting neonates, ii) clinical parameters of transported neonates at arrival, and iii) outcomes such as survival/mortality and NICU stay (before and after skill-based educational intervention).

**Methods:**

We conducted a single-arm intervention study (pre-and post) over 18 months. We assessed the perceptions and practices of 77 ambulance personnel on neonatal transport pre- versus post-intervention. Checklists assessed ambulance equipment availability/usage in both phases. We compared clinical parameters and outcomes of transported neonates between the pre-intervention (n=62) and post-intervention (n=53) phases. We analyzed data using SPSS version 25.

**Results:**

Post-intervention, there was a significant reduction in the levels of hypothermia (p < 0.001), hypoglycemia (p=0.010), and prolonged capillary refill time (p=0.042), along with improvement in the use of intravenous fluids (p <0.001), a reduction in the positivity of umbilical swab growth (p=0.002) and in the duration of NICU stay (p = 0.001), significant improvement *(*p < 0.001) in the perceptions/practices of ambulance personnel towards neonatal transport. There was an improvement in the ambulance equipment availability/usage post-intervention.

**Conclusions:**

The perceptions and practices of the ‘108’ ambulance towards transporting neonates had significantly improved post-educational intervention. Further, a significant decrease in hypothermia, hypoglycemia, and duration of NICU stay was seen in neonates transported post-intervention.

## Introduction

Neonatal transport is an evolving and challenging concept in the Indian scenario. ^1^ Challenges are primarily due to constrained and non-uniform distribution of health care facilities and inadequate transport systems. Though in-utero transport is best, we cannot predict preterm delivery/prospective perinatal problems. Thus arises the need for a dedicated transport facility to an apt well-equipped health care centre. ^2^^–^^4^

Among the ambulances, the ‘108’ ambulance service of Emergency Management and Research Institute (EMRI) ^5^^,^^6^ is a public-private partnership between Government and private emergency medical services. The number ‘108’ stands for the toll-free emergency telephone number across various states in India. They provide services for the public free of cost, are time-trusted, frequently used, and available across multiple districts of Karnataka. Specialised neonatal ambulances from EMRI are available in Tamilnadu and Goa and is not yet having services in Karnataka state. Our study concentrated on only neonates transported by ‘108’ ambulances.

The Indian Government has demonstrated a strong political commitment to lowering newborn mortality. In India, newborn fatalities make up 27% of all neonatal deaths worldwide. The Sustainable Development Goal target 3.2 ^7^^,^^8^ and the Indian Newborn Action Plan (INAP) goal ^9^ of a neonatal mortality rate to 12 or less per 1000 live births by 2030 is yet to be achieved by most of the states in India and is challenging. One of the significant obstacles to achieving this goal is the lack of dedicated neonatal transport. Neonatal survival depends not only on the quality of care delivered to the neonate in the NICU but also on the neonate's condition during NICU admission. ^10^

The golden hour management in neonatal care, stabilization before and during transport, has improved outcomes. ^11^^–^^13^ It is known that transporting sick neonates in specialized transportation with well-assembled and skilled teams can reduce mortality. ^1^^,^^11^^,^^13^ Navjat Shishu Suraksha Karyakram (NSSK), introduced by the Government of India (GOI), also accentuates safe neonatal transport. ^14^

Early identification of babies with altered acute physiology to determine the need for referral, care during transport, and timely therapy at the neonatal intensive care unit (NICU) is known to stop the progression of morbidity, aid recovery, and reduce mortality. ^15^At the same time, ineffectual transport will lead to complications like hypothermia, hypoxia, and hypoglycemia, which can adversely affect neonatal outcomes. Previous studies related to transported neonates have recorded hypothermia between (27-55.3%), ^15^^–^^22^ poor circulation in (8.6-43.42%), ^15^^,^^16^^,^^19^^,^^21^^,^^22^ and hypoglycemia in (7.4 to 35%) ^16^^,^^18^^–^^22^ of neonates. However, facilitated referral in dedicated neonatal ambulances have far less incidences of hypothermia and hypoglycemias. ^1^^,^^11^^,^^13^^,^^23^

Pre-hospital and emergency care should be properly taught to the personnel who work in emergency medical services and accompany patients in ambulances. They should be equipped with the knowledge and abilities needed to act appropriately at the right time in an emergency and to care for a patient until they are transferred to a primary care team. Previous studies have documented varied knowledge, attitudes and practices towards prehospital care and emergency management while transporting patients in ambulances for further care. ^24^^,^^25^

Thus, understanding and rectifying the ambulance personnel's perception/practices towards neonatal transport is essential for effective neonatal outcomes. With this background, we conducted the present study to assess the difference in the perceptions/practices of ‘108’ ambulance personnel towards transporting newborns pre and post skill-based educational interventional training in a coastal city of South India. In addition, we assessed the impact of the intervention on the arrival clinical parameters and clinical outcomes (survival/mortality, NICU duration of stay, blood culture positivity rates) of the neonate's pre- versus post-intervention.

## Methods

### Study design and setting

We conducted a pre and post skill based educational interventional study over 18 months at tertiary neonatal intensive care units attached to a medical college hospital in Southern India. We designed the study to assess the impact of skill-based educational training on ‘108’ ambulance personnel for early newborn care. The participant recruitment and data collection process started from 1 June, 2016.

### Study design

**Figure 1.**
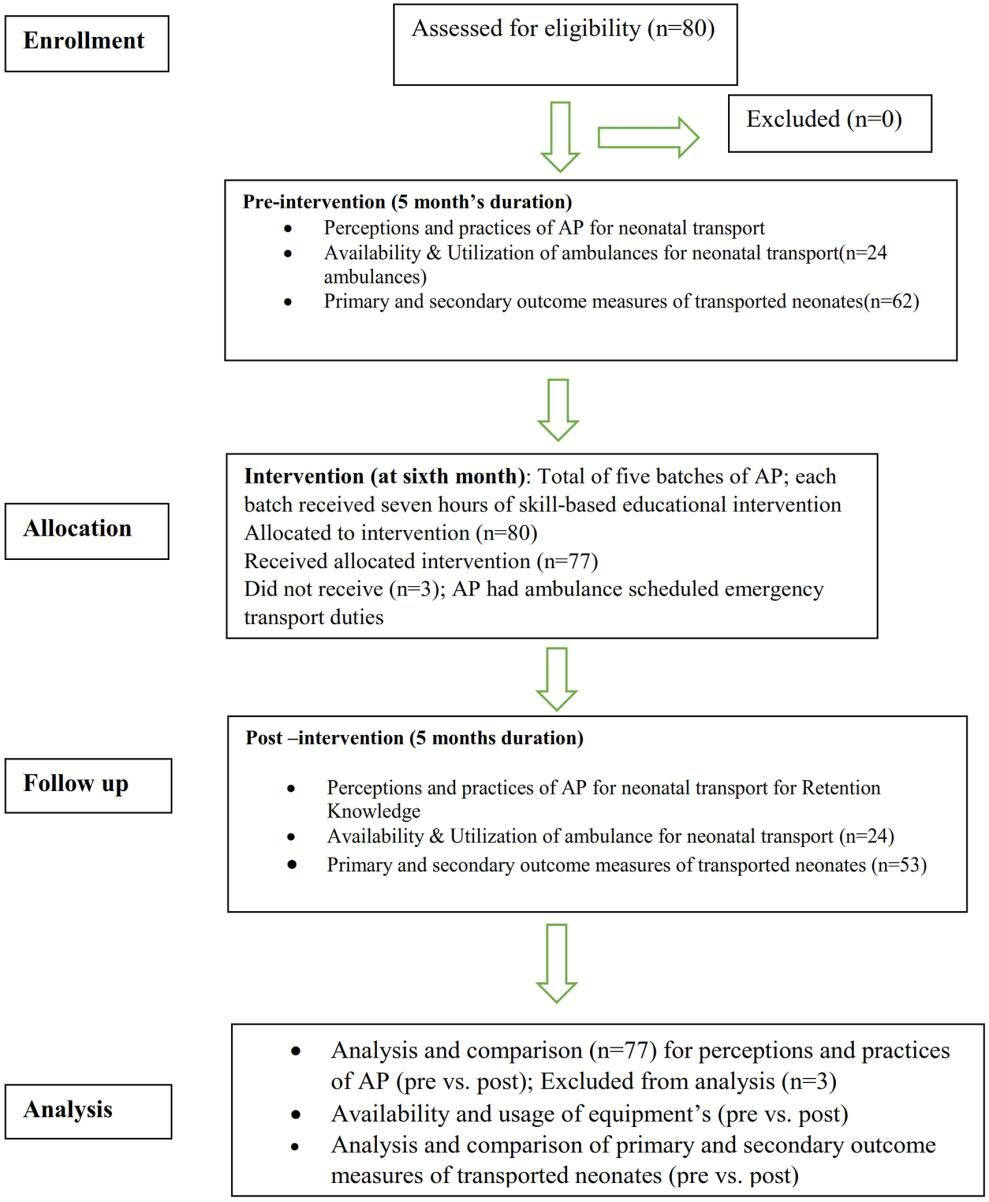
Depicts the study flow according to CONSORT 2010 criteria. ^27^

This research follows the Consolidated Standards of Reporting Trials (CONSORT) statement guidelines.” ^26^ The Reporting guidelines contain a completed CONSORT 2010 checklist. ^27^ Figure 1 depicts the study flow according to CONSORT 2010 criteria. ^27^

### Study participants

It was a time bound study. The study population comprised of

1. The ‘108’ Ambulance personnel (AP - drivers and paramedic staff):

All the ambulance personnel (AP) of the ‘108’ ambulances who transported the neonates to our center during the study period were included for the skill-based educational intervention.

2. The transported neonates to the center by ‘108’ ambulance services:

All the neonates transported only by the ‘108’ ambulances to our center during the study period were included for studying the neonatal outcomes (primary and secondary) measures.

### Ethical Principles

The study was conducted in accordance with the 1964 Declaration of Helsinki, its subsequent revisions, and other relevant ethical guidelines. The institutional ethics committee of Kasturba Medical College, Mangalore, Manipal Academy of Higher education, Karnataka, Manipal, India (IEC KMC MLR 05-16/102, dated May 18, 2016) authorized the study. We took necessary permissions from the hospital authorities and the authorities concerned with the ‘108’ ambulance personnel. We obtained written informed consent from the ‘108’ ambulance staff and the parents/guardians of the newborns to participate in the study and publish the results. (as in *Extended data*). ^27^

We registered the study on 26 March 2018 in the Clinical trial registry of India: CTRI registration number CTRI/2018/03/012830 ( https://ctri.nic.in/Clinicaltrials/login.php).

Although this study was a prospective trial, it allowed us to register the protocol trial even after we initiated the first enrollment of the patient/subject, and this was applicable as per CTRI rules until March 31st, 2018.

However, CTRI announced that from April 1st, 2018, registration will be allowed only prospectively for clinical trials/studies, that is, before the enrolment of the first patient.

### Study objectives

A.
**Perceptions and practices of the ‘108’ ambulance personnel**:
  Perceptions and practices of the ‘108’ ambulance personnel towards recognition, monitoring, and preventive strategies for hypothermia, hypoglycemia, abnormal respiratory rates, heart rates, and perfusion, along with practices of aseptic measures during neonatal transport (pre and post skill based educational intervention).
B.
**To assess outcome measures of transported neonates**:
**Primary outcome measures:** included assessment of clinical parameters at arrival to our center related to
  • Thermoregulation,
  • Blood glucose levels,
  • Vitals (heart rate, respiratory rate, and capillary filling time),
  • Presence of connected IV fluids at arrival, and
  • Umbilical swab colonization.
**Secondary outcome measures**: included assessment of the clinical outcomes of transported neonates admitted to NICU related to
  • Survival/mortality,
  • Neonates discharged against medical advice,
  • Duration of Neonatal Intensive Care Unit (NICU stay),
  • Presence/absence of blood culture positive sepsis.

We compared the primary and secondary outcome measures related to the transported neonates between the pre- and post-intervention phases to assess the impact of the skill-based educational training intervention.

### Data collection

We collected the data using the following tools:

Tools 1, 2 and 3 were filled by the resident duty doctor which includes even the first author

**Tool 1**: A structured questionnaire for ambulance personnel to assess their perceptions and practices towards neonatal transport (as in *Extended data*). ^27^ The questionnaire had two sections: section (A) included personal information of the AP; section (B) included the questions which assessed their perceptions and practices towards temperature regulation, glucose control, vital parameters, and asepsis of the neonates transported.

**Tool 2**: Checklists to assess the availability and utilization of the equipment in the ‘108’ ambulances (as in *Extended data*). ^27^

**Tool 3**: A pre-structured proforma for residents at the arrival center to document the primary and secondary outcome measures of the neonates that get transported (as in *Extended data*). ^27^

We used tools 1,2 and 3 to capture the relevant data both before and after the educational intervention for comparison. Subject experts validated the content of tools 1,2 and 3.

**Clinical tools**: included digital thermometers, one-touch glucometer strips, and umbilical culture swabs. Instead of specific neonatal probes, adult probes were used for assessing continuous vitals during transport; thus, oxygen saturation levels were not included as a parameter in our study.

### For neonatal primary and secondary outcome measures

On arrival of neonates at our center, the resident duty doctor-using tool 3 proforma, documented clinical parameters such as temperature, blood glucose, vitals, and the presence of connected intravenous (IV) fluids on the transported neonates. Upon reaching the hospital, a digital thermometer was used to record the axillary temperature. The glucometer was used to measure blood sugar levels. Heart rate and SpO2 measurements were taken using a multipara monitor. By manually counting, the respiratory rate was estimated.

Umbilical swabs were cultured onto chocolate agar and MacConkey agar media, incubated at 37 degrees centigrade for 18-24 hours, and organisms grown were identified by biochemical reactions. These neonates were further followed for documentation of secondary outcome measures on tool 3 proforma by the first author. The clinical parameters and outcomes of transported neonates documented pre- and post-intervention were compared. The study flow of methodology is depicted in Figure 1.

### Operational definitions

1. Hypothermia - axillary temperature of less than 36.5-degree centigrade
2. Hypoglycemia - blood sugar of less than 50mg/dl
3. Prolonged capillary filling time (CFT) - CFT of more than 3 seconds
4. Tachycardia - heart rate ≥ 160 beats per minute
5. Bradycardia - heart rate ≤ 100 beats per minute
6. Tachypnea - respiratory rate of more than 60 cycles per minute.

Neonatal sex was determined by external examination of body characteristics (external genitalia examination).

### Intervention

We divided the ‘108’ ambulance personnel into five batches for the skill-based educational intervention program ( Table 1). Each set was given skill-based academic training as per NRP guidelines for seven hours each, on five separate days, by a team of pediatricians (first three authors) in their local language. We stressed the following concepts as per neonatal resuscitation program (NRP) guidelines ^28^ during the skill-based education training in the form of theoretical knowledge, video-based teaching, and hands-on training to the ambulance personnel:

**Table 1.** Skill-based educational intervention training program for ambulance personnel.

Sl no	*Skill- based educational intervention training program for ambulance personnel*
1.	Hand washing skills, use of sterile gloves
2.	Basic NRP training ▪ Drying, positioning, suctioning, stimulation, repositioning ▪ Selection of appropriate size mask for bag and mask ventilation ▪ Hands-on training in providing a correct method of positive pressure ventilation (PPV) ▪ Observing adequate chest rise. ▪ Ventilator corrective steps
3.	Keeping baby dry and warm
4.	Transport with clean, covered clothing
5.	Secure and check IV-line functioning
6.	Glucose/Temperature monitoring during transport
7.	Regular Sanitization of ambulance and transport trolley
8.	Equipment availability and usage

After five months of intervention, we assessed the retention capacity of skills among the ambulance personnel using the tool 1 questionnaire and the equipment availability/usage using tool 2 checklists.

### Statistical analysis

We analyzed the collected data by IBM Corp. Released 2017. IBM SPSS Statistics for Windows, Version 25.0. Armonk, NY: IBM Corp. We used the Chi-square and Fischer exact P tests to compare the primary and secondary outcome measures. To assess the ambulance personnel's knowledge, we calculated a mean score and used paired t-tests to compare pre- and post-intervention scores.

## Results

### Primary and secondary outcome measures of transported neonates

The number of neonates transported in the pre-intervention and the post-intervention phases were 62 (29 females and 33 males) and 53 (23 females and 30 males), respectively. ^27^ The distance travelled by the 108 ambulances for neonatal transport varied between 0.5 km to two hundred kms: duration of transport ranging between 10 minutes to 360 minutes. Mean birthweight of transported neonates was 2.01 kgs and 2.04 kgs in pre vs post intervention phases. Most neonatal cases referred were preterm neonates (nearly 30%); neonatal sepsis in 25.8 % & 22.6%, and respiratory distress in 13% & 18.9% of neonates in the pre- and post-intervention phases, respectively. Pediatric surgical cases accounted for 6.4% and 7.5% in the pre- and post-intervention phases.

A comparison of the clinical parameters of transported neonates in the pre- versus post-intervention phases ( Table 2) demonstrated a significant reduction in hypothermia (p< 0.001), hypoglycemia (p = 0.010), prolonged capillary refill time (p = 0.042), and a significant parallel improvement in the use of intravenous fluids (p < 0.001) post-intervention ( Table 2). The vital parameters such as heart rate and respiratory rate had shown improvement in percentages of normal vitals post intervention; however, the change was not significant statistically. The spectrum of organisms that grew from the umbilical swab had shown a significant reduction (p=0.002) from 42% (pre-intervention) to 15.1% post-intervention. Staphylococcus aureus was the predominant organism colonizing the umbilical stump in both phases, with the colonization decreasing from 32.2% to 11.3% post-intervention.

**Table 2.** Clinical Parameters of Neonates at Arrival to Centre (pre-intervention versus post-intervention).

Variables	Interpretation	Pre-Intervention Phase (n = 62)	Post Intervention Phase (n = 53)	P-Value
Heart rate	Normal	37 (59.7%)	42 (49.2%)	0.074
	Tachycardia	19 (30.6%)	9 (17%)	
	Bradycardia	6 (9.7%)	2 (3.8%)	
Respiratory rate	Normal	42 (67.7%)	44 (83%)	0.169
	Tachypnea	15 (24.2%)	7 (13.2%)	
	Apnea	5 (8.1%)	2 (3.8%)	
Capillary Filling Time	< 3 seconds	33 (46.8%)	38 (71.7%)	**0.042** *
	>3 seconds	29 (53.2%)	15 (28.3%)	
Hypothermia	Yes	30 (48.4%)	9 (17%)	**< 0.001** *
	No	32 (51.6%)	44 (83%)	
Hypoglycaemia	Yes	24 (38.7%)	9 (16.9%)	**0.010** *
	No	38 (61.3%)	44 (83.1%)	
Intravenous fluids at admission	Yes	18 (29%)	37 (69.8%)	**< 0.001** *
	No	44 (71%)	16 (30.2%)	
Umbilical swab colonisation	Growth present	26(42%)	8(15.1%)	**0.002** *
	No growth	36 (58%)	45 (84.9%)	

* Significant at < 0.05.

The clinical outcomes of transported neonates ( Table 3) showed a significant reduction in the duration of NICU stay (p = 0.001) post-intervention. Mortality had decreased post-intervention to 9.43% from 14.5%; however, the difference was not statistically significant. There was a reduction in blood culture growth the intervention to 15.1% from 24.2%; however, it was not statistically significant. The yield of staphylococcus growth in blood cultures post-intervention had reduced to 25 % from 33%.

**Table 3.** Clinical Outcomes of the Neonates Transported Pre-intervention versus post-intervention phases.

Outcome Measures	Pre-Intervention phase (n=62)	Post Intervention phase (n=53)	P-Value
**Duration of NICU stay (days)**			**0.001** *
< 5	15 (24.2%)	17 (32.1%)	
5-10	20 (32.3%)	12 (22.6%)	
11-20	25 (40.3%)	10 (18.9%)	
>20	2 (3.2%)	14 (26.4%)	
**Final Outcome of neonates**			0.372
Discharged	49 (79.03%)	41 (77.36%)	
Mortality	9 (14.52%)	5 (9.43%)	
Leave against medical advice	4 (6.45%)	7 (13.21%)	
**Blood Culture Growth**			0.224
Growth present	15 (24.2%)	8 (15.1%)	
No Growth	47 (75.8%)	45 (84.9%)	

* Significant at < 0.05.

### Perceptions and practices of ambulance personnel towards neonatal transport

About 80 ambulance personnel got involved in the pre-intervention, while 77 were in post-intervention because of their ambulance duties. Hence, for the perceptions and practices assessment towards neonatal transport, we finally included 77 ambulance personnel.

There was a significant improvement (p < 0.001) in the mean scores of perceptions and practices of ambulance personnel (retention capacity) in the post-intervention versus pre-intervention phase related to different domains in the questionnaire ( Table 4).

**Table 4.** Assessment of ambulance personnel's perceptions and practices toward neonatal transport (pre-intervention versus post-intervention phases) (n=77).

Questions related to domains	Pre-Intervention score Mean ± SD	Post- Intervention score Mean ± SD	P-value
Temperature regulation	25.94 ± 21.36	44.55 ± 23.94	**< 0.001** *
Glucose homeostasis	18.60 ± 17.50	46.27 ± 18.83	**< 0.001** *
Respiration	20.45 ± 18.46	47.73 ± 25.39	**< 0.001** *
Circulation	48.45 ± 25.10	85.26 ± 23.27	**< 0.001** *
Sepsis prevention	39.35 ± 16.85	67.05 ± 17.53	**< 0.001** *

* Significant at < 0.05.

We included 24 ambulances that transported neonates for equipment availability and usage. There was an improvement in the availability and usage of the following equipment: digital thermometer, glucometer, glucometer strips, neonatal masks, and pulse oximeter post-intervention. The use of hand rubs had improved with a reduction in the blood culture growth and umbilical swab colonization.

## Discussion

Neonates' prospects for survival depend not only on the quality and extent of neonatal care offered, but also on the state of the newborn at admission. We studied variations pre versus post educational intervention in i) newborns' arrival clinical parameters, ii) clinical outcomes, iii) ambulance crew perspectives and practices toward neonatal transport, and iv) the availability and use of ambulance equipment.

Most neonates transported in pre- and post-intervention phases of the study were for preterm care and their issues. This observation is akin to other studies done in developing countries. ^2^^,^^15^^,^^23^^,^^29^ After intervention hypothermia and hypoglycemia reduced from 48.4% to 17% and 38.7% to 16.9%, respectively. Neonatal hypoperfusion reduced to 28.3% and IV fluid administration significantly improved from 29% to 69.8% post-intervention. Different studies on neonatal transport show hypothermia in 27% to 55.3%, ^15^^–^^22^ and hypoglycemia in 7.4 to 35 %. ^16^^,^^18^^–^^22^ Hypoperfusion was seen in 8.6 to 43.42% ^15^^,^^16^^,^^19^^,^^21^^,^^22^ newborns in other studies.

The significant reduction in the incidence of hypothermia and hypoglycemia post-intervention is similar to the study by Kaushal et al., which was done in two phases (before versus after training). ^30^ Kaushal *et al*., ^30^ adopted crew training by the STABLE neonatal education program, which has six assessment care modules related to Sugar, Temperature, Airway, Blood pressure, Lab work, and Emotional support; however, in our study, training was as per NRP guidelines. ^28^

Previous research on neonatal transport has demonstrated a substantial correlation between abnormal physiological parameters and newborn mortality. ^19^ Conversely, favorable outcomes were observed when hemodynamic stability was preserved with improved vitals ^31^^,^^32^ throughout transport; these were noticeably higher when a committed, knowledgeable team provided neonatal care. ^1^^,^^11^^,^^13^^,^^23^ The efficiency of dedicated neonatal ambulance services was indicated by the significantly lower rates of hypothermia (2.3% ^23^ & 3.2% ^1^), hypoglycemia (3.2% ^1^ & 4.59% ^23^), and hypoperfusion (3.44% of cases ^23^).

Contrary to other studies, ^17^^,^^22^^,^^23^ our study had lesser mortality rates of transported neonates and a significant reduction in NICU stay duration. This could be because of improved neonatal transport post-intervention and because of different spectrum of cases during both the phases. In addition, the reduction in incidences of hypoglycemia, hypothermia, and abnormal CRT in our study was probably because of i) concurrent improvement in the availability and usage of thermometers and glucostrips, ii) clean clothing for wrapping baby, iii) neonatal masks for supplementing oxygen and iv) transporting newborns with IV access and fluids as indicated.

Despite the intervention, equipment such as neonatal saturation probes, neonatal warmers, and embrace was unavailable. Our study documented the facilities available for transporting neonates by ‘108’ ambulances needs optimization. Upgrading facilities of ‘108’ ambulances and training ambulance personnel would improve outcomes of transported newborns and is similar to findings by Manikyamba et al. ^21^

Post-intervention, the transported neonates in the current study showed significant reduction in umbilical swab colonization growth and in blood culture positivity rates with parallel increase in the hand rubs availability and use. The selection of antibiotics and the cleanliness of referral facilities are two further factors that could have impacted on our study's results.

The mean scores of perceptions and practices of the ambulance personnel in our study had improved in the post-intervention phase with skill-based educational intervention measures. It had shown an impact on primary and secondary outcome measures and the availability and usage of equipment to a certain extent.

The retention knowledge (perception and practices) of ambulance personnel even after six months of intervention along with improvement in the arrival clinical parameters and clinical outcomes at discharge indicates sustainability of the intervention. In addition, capacity-building or reinforcing skills at the primary level in newborn care, recognizing danger signs, implementing early referrals, adopting safe neonatal transport measures, and conducting repeated refresher courses for ambulance personnel would aid in achieving the goal of single-digit NMR. ^23^^,^^33^ We could appropriately use Evidence-based Kangaroo mother care interventions during transport. A Cochrane review ^34^ suggested that cluster trials (comparing groups of hospitals) with specialist teams for neonatal transport could provide better evidence about mortality and morbidity issues.

### Limitations

The current study had confounding factors, which could have caused a result bias. Knowledge bias could be produced using pre-intervention questionnaire surveys and background information to determine ambulance personnel awareness and practices. Factors such as choice of antibiotics administered and variations in prior treatment and referral hospital environment could have affected the blood culture growth, the details of which we did not collect in the study because of feasibility issues. Though preterm cases were the primary referrals, the spectrum of cases that arrived in both phases was varied. Our findings may potentially have been influenced by risk factors for early onset sepsis, thus we recommend that future research should undertake molecular typing of organisms from a variety of locations, such as ambulances and referral clinics, to help determine the main point of infection dissemination. The neonatal clinical parameters at arrival and neonatal outcomes during NICU stay can differ from case to case.

## Conclusions

Education of ambulance personnel on basic newborn resuscitation, asepsis, temperature regulation, and glucose maintenance reduce frequency of hypothermia, hypoglycemia, hypoperfusion, and infection in transported neonates and has an improvement in their perceptions/practices.

### Ethics & consent

The study was conducted in accordance with the 1964 Declaration of Helsinki, its subsequent revisions, and other relevant ethical guidelines. The institutional ethics committee of Kasturba Medical College, Mangalore, Manipal Academy of Higher education, Karnataka, Manipal, India (IEC KMC MLR 05-16/102, dated May 18, 2016) authorized the study. We took necessary permissions from the hospital authorities and the authorities concerned with the ‘108’ ambulance personnel. We obtained written informed consent from the ‘108’ ambulance staff and the parents/guardians of the newborns to participate in the study and publish the results

## Data Availability

Open Scientific Framework: Effect of skill-based educational training for ambulance personnel on neonatal transport for newborn care in coastal south India -A single arm intervention study. https://doi.org/10.17605/OSF.IO/J5GQ7. ^27^ This dataset contains the following underlying data:
• Pre intervention phase data excel sheet. • Post intervention phase data excel sheet. Open Scientific Framework: Effect of skill-based educational training for ambulance personnel on neonatal transport for newborn care in coastal south India -A single arm intervention study. https://doi.org/10.17605/OSF.IO/J5GQ7. ^27^ This dataset contains the following underlying extended data:
• Participant information sheet (ambulance personnel **)**
• Parent/guardian information sheet • Informed consent form for ambulance personnel • Informed consent form for parents/guardians of neonates • Perceptions and practices questionnaire (ambulance personnel) • Checklist questionnaire for equipment availability and usage • Questionnaire for Primary and Secondary Outcome measures of neonates Open Scientific Framework: CONSORT 2010 checklist for ‘Effect of skill-based educational training for ambulance personnel on neonatal transport for newborn care in coastal south India -A single arm intervention study’. https://doi.org/10.17605/OSF.IO/J5GQ7. ^27^ Data are available under the terms of the Creative Commons Attribution 4.0 International license (CC-BY 4.0).
