# Effect of skill-based educational training for ambulance personnel on neonatal transport for newborn care in coastal South India – a single arm intervention study

**DOI:** 10.12688/f1000research.150058.2

**Published:** 2025-01-02

**Authors:** Santosh Kalyan, Sowmini Padmanabh Kamath, Subhodh Shetty S, Ramesh Holla, Leslie Lewis, Harsha Lashkari P, Suchitra Shenoy M, Shantharam Baliga B

**Affiliations:** 1 Department of Pediatrics, Kasturba Medical College Mangalore, Manipal Academy of Higher Education, Karnataka, Manipal, 576104, India; 2 Department of Neonatology, Kasturba Medical College, Manipal Academy of Higher Education, Karnataka, Manipal, 576104, India; 3 Department of Community Medicine, Kasturba Medical College Mangalore, Manipal Academy of Higher Education, Karnataka, Manipal, 576104, India; 4 Department of Pediatrics, Kasturba Medical College, Manipal Academy of Higher Education, Karnataka, Manipal, 576104, India; 5 Department of Microbiology, Kasturba Medical College Mangalore, Manipal Academy of Higher Education, Karnataka, Manipal, 576104, India

**Keywords:** Ambulances, Checklist, Hypothermia, Hypoglycemia, Infant, Intensive care units, Neonatal, Newborn

## Abstract

**Background:**

Quality of neonatal transport services may impact outcomes. We assessed the effect of skill-based education of ambulance personnel (AP) on outcomes of transported neonates.

**Methods:**

We conducted a single-arm intervention study (pre and post) over 18 months. We assessed the perceptions and practices of AP on neonatal transport along with ambulance equipment availability/usage. We compared neonatal clinical vital parameters at arrival and clinical outcomes from Neonatal Intensive Care Unit (NICU) pre versus post intervention. We analysed data using SPSS version 25.

**Results:**

Of 77 AP receiving education, there was significant *(*p < 0.001) improvement in the perceptions/practices towards temperature regulation (44.55 ± 23.94 *vs*. 25.94 ± 21.36), glucose homeostasis (46.27 ± 18.83 *vs.* 18.60 ± 17.50), maintaining asepsis (67.05 ± 17.53 *vs.* 39.35 ± 16.85), supporting airway (47.73 ± 25.39), circulation (85.26 ± 23.27 *vs.* 48.45 ± 25.10), along with ambulance equipment availability/usage postintervention. Of 53 neonates studied post intervention, there was a significant reduction in hypothermia (17% *vs.* 48.4%, p < 0.001), hypoglycemia (16.9% *vs.* 38.7%, p=0.010), and prolonged capillary refill time (71.7% *vs.* 46.8%, p=0.042), improvement in the use of intravenous fluids (69.8% *vs.* 29%, p <0.001), a reduction in growth from umbilical swabs (15.1% *vs.* 42%, p=0.002) and duration of NICU stay (p = 0.001).

**Conclusions:**

After educational intervention there was significant improvement in the perceptions and practices of ‘108’ ambulance personnel towards transporting neonates and a significant decrease in hypothermia, hypoglycemia, and duration of NICU stay with improvement in maintenance of circulation and asepsis in neonates.

## Introduction

The first month of life is the most critical time for a child’s survival and demands higher-quality intrapartum and neonatal care. An essential component of newborn care is neonatal transport. Worldwide, neonatal mortality has decreased from 5.0 million to 2.3 million deaths between 1990 and 2022. Approximately 6500 newborns die every day, accounting for 47% of the deaths of children under the age of five. Preterm birth, infections, birth asphyxia, and birth abnormalities cause most neonatal deaths but conditions during transfer to a higher center, may also contribute to mortality and morbidity. The neonatal mortality rate globally is 17/1000 live births, whereas in India, it is 23/1000 live births. ^1, 2^

Neonatal transport is an evolving and challenging concept in the Indian context, ^3^ due to the constrained and nonuniform distribution of health care facilities and inadequate systems. Although in-utero transport is best, we cannot predict preterm delivery/prospective perinatal problems. Thus the need arises for a dedicated transport facility to an apt well-equipped health care center. ^4– 6^ Among the ambulances, the ‘108’ ambulance service of the Emergency Management and Research Institute (EMRI) ^7^^,^^8^ is a public–private partnership with ‘108’ being the toll-free emergency telephone number across various Indian states. They provide services for the public free of cost, are time-trusted, frequently used, and available across multiple districts of Karnataka but specialized neonatal ambulances from EMRI are available only in Tamilnadu and Goa. Our study focused on only neonates transported by ‘108’ ambulances.

The Indian government has demonstrated a strong political commitment to lowering newborn mortality. In India, newborn fatalities constitute 27% of all neonatal deaths worldwide. The Sustainable Development Goal target 3.2 ^9^^,^^10^ and the Indian Newborn Action Plan (INAP) goal ^11^ of a neonatal mortality rate of 12 or fewer per 1000 live births by 2030 is yet to be achieved by most states in India and is challenging. One of the significant obstacles to achieving this goal is the lack of dedicated neonatal transport. Neonatal survival depends not only on the quality of care delivered to the neonate in the NICU but also on the neonate’s condition during NICU admission. ^12^

The golden hour of management in neonatal care, stabilization before and during transport, has improved outcomes. ^13– 15^ Transporting sick neonates via specialized transportswith well-assembled and skilled teams can reduce mortality. ^3^^,^^13, 15^ Navjat Shishu Suraksha Karyakram (NSSK), introduced by the Government of India (GOI), also highlights safe neonatal transport. ^16^

Early identification of babies with altered acute physiology to determine the need for referral, care during transport, and timely therapy at the neonatal intensive care unit (NICU) is known to stop the progression of morbidity, aid recovery, and reduce mortality. ^17^ Moreover, ineffectual transport can lead to complications such ashypothermia, hypoxia, and hypoglycemia, which can adversely affect neonatal outcomes. Previous studies related to transported neonates have recorded hypothermia between 27 to 55.3%, ^17– 24^ poor circulation in 8.6 to 43.42%, ^17, 18, 21, 23, 24^ and hypoglycemia in 7.4 to 35% ^18, 20– 24^ of neonates. However, facilitated referral in dedicated neonatal ambulances have far lower incidences of hypothermia and hypoglycemias. ^3, 13, 15, 25^

Thus, understanding and rectifying the ambulance personnel’s perception/practices towards neonatal transport is essential for effective neonatal outcomes. We studied skills of the ‘108’ ambulance personnel towards recognition, monitoring, and preventive strategies for hypothermia, hypoglycemia, abnormal respiratory rates, heart rates, and perfusion, practices of aseptic measures during neonatal transport before and after a skill based educational intervention. In addition, we assessed the impact of the intervention on the arrival clinical parameters (e.g. vitals, temperature, blood glucose, etc.) and clinical outcomes (survival/mortality, NICU duration of stay, blood culture positivity rates).

## Methods

### Study design, setting and ethics

This research follows the “Consolidated Standards of Reporting Trials (CONSORT) statement guidelines.” ^26^ The Reporting guidelines contain a completed CONSORT 2010 checklist. ^27^ Figure 1 depicts the study flow according to CONSORT 2010 criteria. ^27^ This single-arm intervention trial (pre- versus postintervention) was designed to assess the impact of skill-based educational training on ‘108’ ambulance personnel for early newborn care over 18 months at a tertiary neonatal intensive care unit attached to a medical college hospital in southern India.

The participant recruitment and data collection process started on 1 June, 2016.

The study was conducted in accordance with the 1964 Declaration of Helsinki, its subsequent revisions, and other relevant ethical guidelines. The institutional ethics committee of Kasturba Medical College, Mangalore, Manipal Academy of Higher education, Karnataka, Manipal, India (IEC KMC MLR 05-16/102, dated May 18, 2016) authorized the study. We took necessary permissions from the hospital authorities and the authorities concerned with the ‘108’ ambulance personnel. We obtained written informed consent from the ‘108’ ambulance staff and the parents/guardians of the newborns to participate in the study and publish the results (as in *Extended data*). ^27^

We registered the study on 26 March 2018 in the Clinical trial registry of India: CTRI registration number CTRI/2018/03/012830 ( https://ctri.nic.in/Clinicaltrials/login.php).

Although this study was a prospective trial, it allowed us to register the protocol trial even after we initiated the first enrollment of the patient/subject, and this was applicable as per CTRI rules until March 31st, 2018.

However, CTRI announced that from April 1st, 2018, registration will be allowed only prospectively for clinical trials/studies, that is, before the enrolment of the first patient.

Participant recruitment:

The inclusion criterion for the study population was as follows:

1. The neonates transported to the center by ‘108’ ambulance services:
All the consecutive neonates transported only by the ‘108’ ambulances to our center during the study period were included for the neonatal outcomes (primary and secondary) measures.
2. The neonates transported to the center by ‘108’ ambulance services:
All the consecutive neonates transported only by the ‘108’ ambulances to our center during the study period were included for the neonatal outcomes (primary and secondary) measures.

***Exclusion criteria:*** The AP and the parents/guardians who expressed unwillingness to be a part of the research were excluded. The AP’s who could not attend the educational intervention program were excluded.

### Data collection

We collected the data via the following tools:

Tools 1, 2 and 3 were filled by the resident duty doctor.

**Tool 1**: A structured questionnaire for ambulance personnel to assess their perceptions and practices towards neonatal transport (as in *Extended data*). ^27^ The questionnaire had two sections: section (A) included personal information of the AP; section (B) included the questions that assessed perceptions and practices related to temperature regulation, glucose control, vital parameters, and asepsis of the neonates transported.

**Tool 2**: Checklists to assess the availability and utilization of the equipment in the ‘108’ ambulances (as in *Extended data*). ^27^

**Tool 3**: A pre-structured proforma for residents at the arrival center to document the primary and secondary outcome measures of the neonates that are transported (as in *Extended data*). ^27^

We used tools 1,2 and 3 to capture the relevant data both before and after the educational intervention for comparison. Subject experts validated the content of tools 1,2 and 3.

**The clinical tools** used included digital thermometers, one-touch glucometer strips, and umbilical culture swabs. Instead of specific neonatal probes, adult probes were used for assessing continuous vitals during transport; thus, oxygen saturation levels were not included as a parameter in our study.

On the arrival of neonates at our center, the resident duty doctor-using tool 3 proforma, documented clinical parameters such as temperature, blood glucose, vitals, and the presence of connected intravenous (IV) fluids on the transported neonates. Upon reaching the hospital, a digital thermometer was used to record the axillary temperature. A glucometer was used to measure blood sugar levels. Heart rate and SpO _2_ measurements were taken via a multipara monitor. The respiratory rate was estimated via manual counting.

Umbilical swabs were cultured on chocolate agar and MacConkey agar media, incubated at 37 degrees centigrade for 18-24 hours, and organisms grown were identified via biochemical reactions. These neonates were further followed for documentation of secondary outcome measures on tool 3 proforma by the first author. The clinical parameters and outcomes of transported neonates documented pre- and post-intervention were compared. The study flow of methodology is depicted in Figure 1.

### Operational definitions

The clinical physiological vital parameters were defined as follows i). Hypothermia - axillary temperature of less than 36.5 degree centigrade ii). Hypoglycemia - blood sugar of less than 50 mg/dl, iii). Prolonged capillary filling time (CFT) - CFT of more than 3 seconds, iv). Tachycardia - heart rate ≥ 160 beats per minute, v). Bradycardia - heart rate ≤ 100 beats per minute vi). Tachypnea - respiratory rate > 60 cycles per minute. Neonatal sex was determined by external examination of body characteristics (external genitalia examination). ^28, 29^

### Intervention

We divided the ‘108’ ambulance personnel into five batches for the skill-based educational intervention program ( Table 1). Each set was given skill-based academic training as per NRP guidelines for seven hours each, on five separate days, by a team of pediatricians (first three authors) in their local language. We stressed the following concepts as per neonatal resuscitation program (NRP) guidelines ^30^ during skill-based education training in the form of theoretical knowledge, video-based teaching, and hands-on training for ambulance personnel:

**Table 1.** Skill-based educational intervention training program for ambulance personnel.

Sl no	*Skill-based educational intervention training program for ambulance personnel*
1.	Hand washing skills, use of sterile gloves
2.	Basic NRP training ▪ Drying, positioning, suctioning, stimulation, repositioning ▪ Selection of appropriate size mask for bag and mask ventilation ▪ Hands-on training in providing a correct method of positive pressure ventilation (PPV) ▪ Observing adequate chest rise. ▪ Ventilator corrective steps
3.	Keeping baby dry and warm
4.	Transport with clean, covered clothing
5.	Secure and check IV-line functioning
6.	Glucose/Temperature monitoring during transport
7.	Regular Sanitization of ambulance and transport trolley
8.	Equipment availability and usage

After five months of intervention, we assessed the retention capacity of skills among the ambulance personnel via the tool 1 questionnaire and the equipment availability/usage via tool 2 checklists.

**Outcome measures**

1. Outcomes in AP - Perceptions and practice
2. Outcomes in neonates

a). *Primary outcome measures*: included assessment of clinical parameters at arrival to our center related to

• Thermoregulation,
• Blood glucose levels,
• Vitals (heart rate, respiratory rate, and capillary filling time),
• Presence of connected IV fluids at arrival, and
• Umbilical swab colonization.

b). *Secondary outcome measures*: included assessment of the clinical outcomes of transported neonates admitted to NICU related to

• Survival/mortality,
• Neonates discharged against medical advice,
• Duration of NICU stay,
• Presence/absence of blood culture positive sepsis.

### Statistical analysis

We analyzed the data collected by IBM Corp. Released 2017. IBM SPSS Statistics for Windows, Version 25.0. Armonk, NY: IBM Corp. We used the Chi-square and Fischer exact P test to compare the primary and secondary outcome measures. To assess the ambulance personnel's knowledge, we calculated a mean score and used paired t-tests to compare pre- and post-intervention scores.

## Results

**Figure 1.**
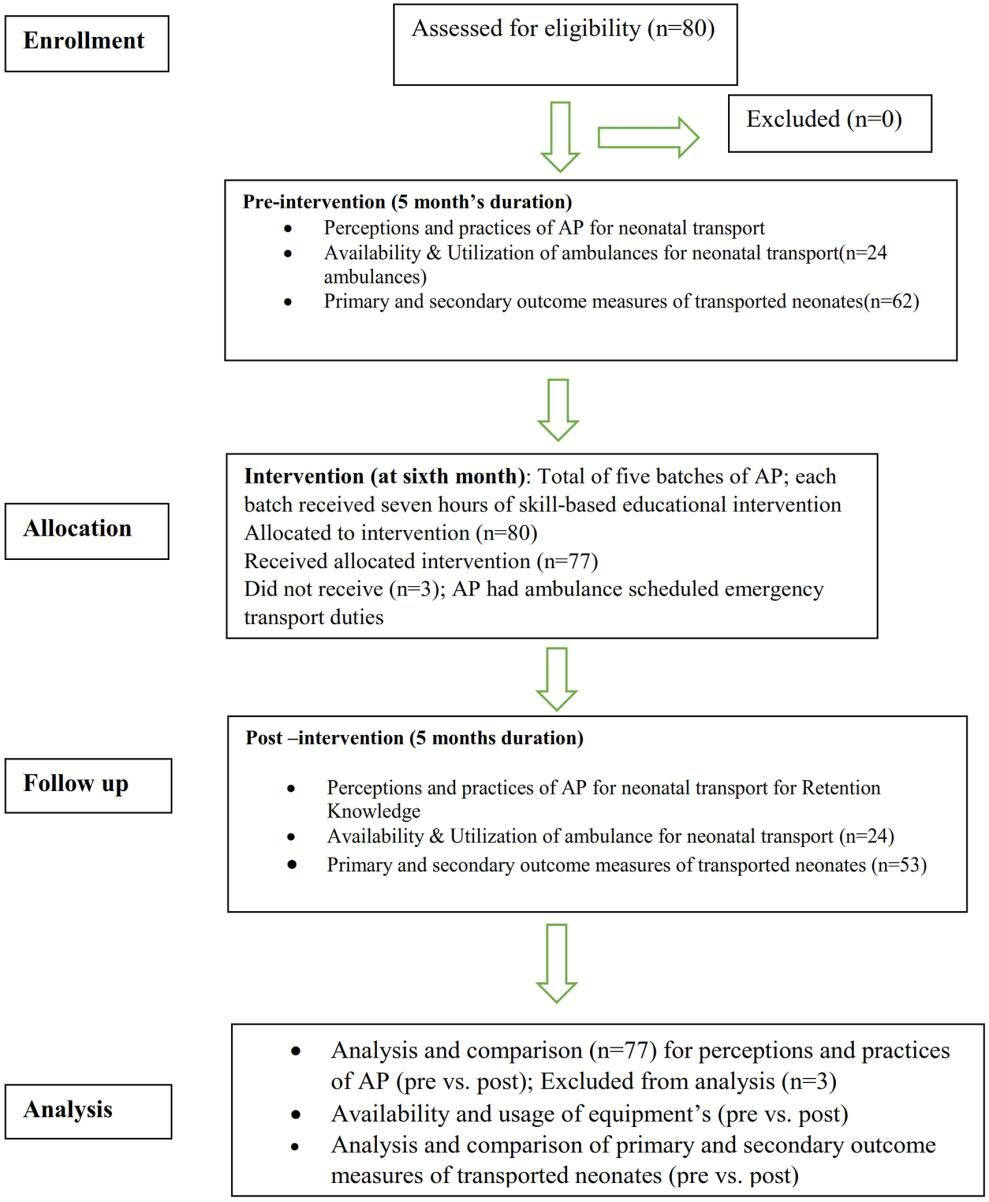
Depicts the study flow according to CONSORT 2010 criteria. ^27^

### Baseline characteristics of AP and transported neonates

The numbers of neonates transported in the pre-intervention and the post-intervention phases were 62 (29 females and 33 males) and 53 (23 females and 30 males), respectively. ^27^ The distance travelled by the 108 ambulances for neonatal transport varied between 0.5 km and two hundred kms: duration of transport ranging between 10 minutes and 360 minutes. Mean birthweight of transported neonates was 2.01 kgs and 2.04 kgs in the pre- and postintervention phases. Most neonatal cases referred to were preterms (nearly 30%); neonatal sepsis in 25.8 % & 22.6%, and respiratory distress in 13% & 18.9% of neonates in the pre- and postintervention phases, respectively. Pediatric surgical cases accounted for 6.4% and 7.5% in the pre- and postintervention phases respectively. For the 80 APs recruited, data on 77 were available for both before and after the intervention. Educational qualifications of 77 APs included 52 matriculates, 23 preuniversity, and two graduates.

### Primary and secondary outcome measures of transported neonates

After intervention ( Table 2), there was significant reduction in hypothermia (p < 0.001), hypoglycemia (p = 0.010), prolonged capillary refill time (p = 0.042), and a significant parallel improvement in the use of intravenous fluids (p < 0.001) in transported neonates. There was a trend towards improvement of vital parameters such as heart rate and respiratory rate postintervention which was not statistically significant. The growth of the organisms from the umbilical swabs significantly decreased (p = 0.002) from 42% to 15.1% and the predominant organism viz. Staphylococcus aureus decreased from 32.2% to 11.3% postintervention.

**Table 2.** Clinical Parameters of Neonates at Arrival to Centre (pre-intervention versus post-intervention).

Variables	Interpretation	Pre-Intervention Phase (n = 62)	Post Intervention Phase (n = 53)	P-Value
Heart rate	Normal	37 (59.7%)	42 (49.2%)	0.074
	Tachycardia	19 (30.6%)	9 (17%)	
	Bradycardia	6 (9.7%)	2 (3.8%)	
Respiratory rate	Normal	42 (67.7%)	44 (83%)	0.169
	Tachypnea	15 (24.2%)	7 (13.2%)	
	Apnea	5 (8.1%)	2 (3.8%)	
Capillary Filling Time	< 3 seconds	33 (46.8%)	38 (71.7%)	**0.042** *
	>3 seconds	29 (53.2%)	15 (28.3%)	
Hypothermia	Yes	30 (48.4%)	9 (17%)	**< 0.001** *
	No	32 (51.6%)	44 (83%)	
Hypoglycaemia	Yes	24 (38.7%)	9 (16.9%)	**0.010** *
	No	38 (61.3%)	44 (83.1%)	
Intravenous fluids at admission	Yes	18 (29%)	37 (69.8%)	**< 0.001** *
	No	44 (71%)	16 (30.2%)	
Umbilical swab colonisation	Growth present	26(42%)	8(15.1%)	**0.002** *
	No growth	36 (58%)	45 (84.9%)	

* Significant at < 0.05.

There was a significant reduction in the duration of NICU stay (p = 0.001) postintervention. There was a nonsignificant decrease in mortality from 14.5% to 9.43%, growth in blood culture from 24.2% to 15.1%, and yield of staphylococcus in blood cultures from 33% to 25 % post-educational intervention ( Table 3).

**Table 3.** Clinical Outcomes of the Neonates Transported Pre-intervention versus post-intervention phases.

Outcome Measures	Pre-Intervention phase (n=62)	Post Intervention phase (n=53)	P-Value
**Duration of NICU stay (days)**			**0.001** *
< 5	15 (24.2%)	17 (32.1%)	
5-10	20 (32.3%)	12 (22.6%)	
11-20	25 (40.3%)	10 (18.9%)	
>20	2 (3.2%)	14 (26.4%)	
**Final Outcome of neonates**			0.372
Discharged	49 (79.03%)	41 (77.36%)	
Mortality	9 (14.52%)	5 (9.43%)	
Leave against medical advice	4 (6.45%)	7 (13.21%)	
**Blood Culture Growth**			0.224
Growth present	15 (24.2%)	8 (15.1%)	
No Growth	47 (75.8%)	45 (84.9%)	

* Significant at < 0.05.

### Perceptions and practices of ambulance personnel towards neonatal transport

There was a significant improvement (p < 0.001) in the mean scores of perceptions and practices of ambulance personnel (retention capacity) in the post-intervention versus preintervention phase which was related to different domains in the questionnaire ( Table 4).

**Table 4.** Assessment of ambulance personnel's perceptions and practices toward neonatal transport (pre-intervention versus post-intervention phases) (n=77).

Questions related to domains	Pre-Intervention score Mean ± SD	Post- Intervention score Mean ± SD	P-value
Temperature regulation	25.94 ± 21.36	44.55 ± 23.94	**< 0.001** *
Glucose homeostasis	18.60 ± 17.50	46.27 ± 18.83	**< 0.001** *
Respiration	20.45 ± 18.46	47.73 ± 25.39	**< 0.001** *
Circulation	48.45 ± 25.10	85.26 ± 23.27	**< 0.001** *
Sepsis prevention	39.35 ± 16.85	67.05 ± 17.53	**< 0.001** *

* Significant at < 0.05.

### Ambulance equipment availability and usage

Among the 24 ambulances that transported neonates, there was an improvement in the availability and usage of the following equipment: digital thermometer, glucometer, glucometer strips, neonatal masks, and pulse oximeters postintervention. The use of hand rubs had improved with a reduction in the blood culture growth and umbilical swab colonization.

## Discussion

Neonates’ prospects for survival depend not only on the quality and extent of neonatal care offered, but also on the state of the newborn at admission. We studied variations pre versus posteducational intervention in terms of i) newborn arrival clinical parameters, ii) clinical outcomes, iii) ambulance crew perspectives and practices toward neonatal transport, and iv) the availability and use of ambulance equipment.

Most neonates transported in pre- and postintervention phases of the study were for preterm care and their issues. This observation is akin to other studies performed in developing countries. ^4, 17, 25, 31^ After the intervention, the rates of hypothermia and hypoglycemia reduced from 48.4% to 17% and 38.7% to 16.9%, respectively. Neonatal hypoperfusion reduced to 28.3% and IV fluid administration significantly improved it from 29% to 69.8% postintervention. Different studies on neonatal transport have shown that 27% to 55.3%, ^17– 24^ of neonates are hypothermic, and 7.4 to 35 %. ^18, 20- 24^ are hypoglycemic. Hypoperfusion was observed in 8.6 to 43.42% ^17, 18, 21, 23, 24^ of newborns in other studies.

The significant reduction in the incidence of hypothermia and hypoglycemia postintervention is similar to that reported in the study by Kaushal et al., which was performed in two phases (before versus after training). ^32^ Kaushal *et al*., ^32^ adopted crew training via the STABLE neonatal education program, which has six assessment care modules related to Sugar, Temperature, Airway, Blood pressure, Lab work, and Emotional support; however, in our study, training was performed in accordance with the NRP guidelines. ^30^

Research on neonatal transport has demonstrated a substantial correlation between abnormal physiological parameters and newborn mortality. ^20, 21, 33^ Conversely, favorable outcomes were observed when hemodynamic stability was preserved with improved vitals ^33, 34^ throughout transport; these outcomes were noticeably greater when a committed, knowledgeable team provided neonatal care. ^3, 13, 15, 25^ The efficiency of dedicated neonatal ambulance services was indicated by the significantly lower rates of hypothermia (2.3% ^25^ & 3.2% ^3^), hypoglycemia (3.2% ^3^ & 4.59% ^25^), and hypoperfusion (3.44% of cases ^25^).

Contrary to other studies, ^19, 24, 25^ our study had lower mortality rates of transported neonates and a significant reduction in NICU stay duration. This could be because of improved neonatal transport postintervention and because of different spectra of cases during both phases. In addition, the reduction in incidence rates of hypoglycemia, hypothermia, and abnormal CRT in our study was probably because of i) concurrent improvements in the availability and usage of thermometers and glucostrips, ii) clean clothing for wrapping baby, iii) neonatal masks for supplementing oxygen and iv) transporting newborns with IV access and fluids as indicated.

Despite the intervention, equipment such as neonatal saturation probes, neonatal warmers, and embrace was unavailable. Our study documented the facilities available for transporting neonates by ‘108’ ambulance needs optimization. Upgrading facilities of ‘108’ ambulances and training ambulance personnel would improve outcomes of transported newborns and is similar to findings by Manikyamba et al. ^23^

Postintervention, the transported neonates in the current study showed significant reductions in umbilical swab colonization growth and in blood culture positivity rates, with parallel increases in hand rub availability and use. The selection of antibiotics and the cleanliness of referral facilities are two further factors that could have impacted our study’s results.

The mean scores of the perceptions and practices of the ambulance personnel in our study had improved in the postintervention phase with skill-based educational intervention measures. It has been shown to have an impact on primary and secondary outcome measures and the availability and usage of equipment to a certain extent.

The retention knowledge (perception and practices) of ambulance personnel even after six months of intervention, along with improvement in the arrival clinical parameters and clinical outcomes at discharge indicates the sustainability of the intervention. In addition, capacity-building or reinforcing skills at the primary level in newborn care, recognizing danger signs, implementing early referrals, adopting safe neonatal transport measures, and conducting repeated refresher courses for ambulance personnel would aid in achieving the goal of single-digit NMR. ^25, 35^ We could appropriately use evidence-based Kangaroo mother care interventions during transport. A Cochrane review ^36^ suggested that cluster trials (comparing groups of hospitals) with specialist teams for neonatal transport could provide better evidence about mortality and morbidity issues.

### Limitations

The current study had confounding factors, which could have caused a bias in the results. Knowledge bias could be produced via the use of preintervention questionnaire surveys and background information to determine ambulance personnel awareness and practices. Factors such as choice of antibiotics administered and variations in prior treatment and referral hospital environments could have affected the blood culture growth, the details of which we did not collect in the study because of feasibility issues. Although preterm cases were the primary referrals, the spectrum of cases that arrived in both phases varied. Our findings may have been influenced by risk factors for early onset sepsis; thus we recommend that future research should undertake molecular typing of organisms from a variety of locations, such as ambulances and referral clinics, to help determine the main point of infection dissemination. The neonatal clinical parameters at arrival and neonatal outcomes during NICU stay can differ from case to case. We also suggest quality improvement approach will be more useful to address the gaps in similar studies in the future.

## Conclusions

The education of ambulance personnel on basic newborn resuscitation, asepsis, temperature regulation, and glucose maintenance reduces the frequency of hypothermia, hypoglycemia, hypoperfusion, and infection in transported neonates and improves their perceptions/practices.

### Ethics & consent

The study was conducted in accordance with the 1964 Declaration of Helsinki, its subsequent revisions, and other relevant ethical guidelines. The institutional ethics committee of Kasturba Medical College, Mangalore, Manipal Academy of Higher education, Karnataka, Manipal, India (IEC KMC MLR 05-16/102, dated May 18, 2016) authorized the study. We obtained necessary permissions from the hospital authorities and the authorities concerned with the ‘108’ ambulance personnel. We obtained written informed consent from the ‘108’ ambulance staff and the parents/guardians of the newborns to participate in the study and publish the results.

## Data Availability

Open Scientific Framework: Effect of skill-based educational training for ambulance personnel on neonatal transport for newborn care in coastal south India -A single arm intervention study. https://doi.org/10.17605/OSF.IO/J5GQ7. ^27^ This dataset contains the following underlying data:
• Pre intervention phase data excel sheet. • Post intervention phase data excel sheet. Open Scientific Framework: Effect of skill-based educational training for ambulance personnel on neonatal transport for newborn care in coastal south India -A single arm intervention study. https://doi.org/10.17605/OSF.IO/J5GQ7. ^27^ This dataset contains the following underlying extended data:
• Participant information sheet (ambulance personnel **)**
• Parent/guardian information sheet • Informed consent form for ambulance personnel • Informed consent form for parents/guardians of neonates • Perceptions and practices questionnaire (ambulance personnel) • Checklist questionnaire for equipment availability and usage • Questionnaire for Primary and Secondary Outcome measures of neonates Open Scientific Framework: CONSORT 2010 checklist for ‘Effect of skill-based educational training for ambulance personnel on neonatal transport for newborn care in coastal south India -A single arm intervention study’. https://doi.org/10.17605/OSF.IO/J5GQ7. ^27^ Data are available under the terms of the Creative Commons Attribution 4.0 International license (CC-BY 4.0).
