# Supplementary material for: Effect of skill-based educational training for ambulance personnel on neonatal transport for newborn care in coastal South India – a single arm intervention study
Source: F1000Res. 2025 Jan 2;13:767. Originally published 2024 Jul 8. [Version 2] doi: 10.12688/f1000research.150058.2 (PMC11342031; doi:10.12688/f1000research.150058.2)
Supplement: Peer review history

# Reviewer report: Reviewer response for version 2

**DOI:** 10.5256/f1000research.176171.r355414

**Published:** 2025-01-09

**Authors:** A Shashidhar Rao

**Affiliations:** 1 Dept of Neonatology, St Johns Medical College Hospital, Bangalore, Karnataka, India

The authors have satisfactorily addressed  the concerns raised and have modified the manuscript accordingly.

Is the work clearly and accurately presented and does it cite the current literature?

Yes

If applicable, is the statistical analysis and its interpretation appropriate?

Yes

Are all the source data underlying the results available to ensure full reproducibility?

Yes

Is the study design appropriate and is the work technically sound?

Partly

Are the conclusions drawn adequately supported by the results?

Yes

Are sufficient details of methods and analysis provided to allow replication by others?

Yes

Reviewer Expertise:

Neonatology

I confirm that I have read this submission and believe that I have an appropriate level of expertise to confirm that it is of an acceptable scientific standard.

# Reviewer report: Reviewer response for version 1

**DOI:** 10.5256/f1000research.164592.r301765

**Published:** 2024-08-22

**Authors:** Sanoj Karayil Mohammad Ali

**Affiliations:** 1 Division of Neonatology, Department of Pediatrics, Sidra Medicine, Doha, Qatar

This is a clinically relevant interventional study with well-defined methodology and positive findings, which is similar to other studies.

 -Clear working title, which states the study design, setting and population.

 -Objective outcome data along with subjective survey from personnel is appropriate for the study

 -Unsure about the significance of umbilical swab colonization as a surrogate marker for compliance with asepsis measures. There was no significant reduction in blood culture positive rates, which can also be affected by factors other than transport.

 -Limitations of the study and risk of bias has been clearly outlined.

Is the work clearly and accurately presented and does it cite the current literature?

Yes

If applicable, is the statistical analysis and its interpretation appropriate?

Yes

Are all the source data underlying the results available to ensure full reproducibility?

Yes

Is the study design appropriate and is the work technically sound?

Yes

Are the conclusions drawn adequately supported by the results?

Yes

Are sufficient details of methods and analysis provided to allow replication by others?

Yes

Reviewer Expertise:

Neonatology, Respiratory physiology, Neonatal transport, hemodynamics

I confirm that I have read this submission and believe that I have an appropriate level of expertise to confirm that it is of an acceptable scientific standard.

# Response

**Published:** 2024-12-20

**Authors:** Sowmini Padmanabh Kamath

**Affiliations:** Kasturba Medical College, Mangalore, India

1. This is a clinically relevant interventional study with a well-defined methodology and positive findings, which is similar to other studies.

 2. Clear working title, which states the study design, setting, and population.

 3. Objective outcome data along with subjective survey from personnel is appropriate for the study

**Author response: **Thank you

 4. Unsure about the significance of umbilical swab colonization as a surrogate marker for compliance with asepsis measures. There was no significant reduction in blood culture positive rates, which can also be affected by factors other than transport.

**Author response:** Umbilical care is a part of essential newborn care & umbilical artery is an important site for entry of bloodstream infections in newborns. This study was to look for the colonization of the umbilical area but as not a surrogate marker for neonatal sepsis. The correlation between umbilical swabs & blood culture was not a part of the study. As highlighted in the study's limitations section, we agree that factors other than transport could have affected blood culture growth.  

 5. Limitations of the study and risk of bias have been clearly outlined.

**Author response: **Thank you

# Reviewer report: Reviewer response for version 1

**DOI:** 10.5256/f1000research.164592.r301769

**Published:** 2024-07-22

**Authors:** Rakesh Kotha

**Affiliations:** 1 neonatology, Niloufer Hospital, Hyderabad, Telangana, India; 2 Osmania Medical College, Hyderabad, Telengana, India

Yes, great study. Mention who took the data; it is better to be the third person who is not involved in the study. Questionnaire validation to be done and mention who prepared. Sample size justification is needed; a low sample size is a limitation. Reference for sample size is better to add. SPSS country version is better to mention. The cause of attrition is better to mention. Is it a quasi-experimental study? Definitions need citations.What is the significance of statistical errors as pre- and post-test people are different? Ambulance drivers qualifications may affect the internal study result; knowing the data better for comparing other studies. Any novelty? Better to add.

Is the work clearly and accurately presented and does it cite the current literature?

Yes

If applicable, is the statistical analysis and its interpretation appropriate?

Yes

Are all the source data underlying the results available to ensure full reproducibility?

Yes

Is the study design appropriate and is the work technically sound?

Yes

Are the conclusions drawn adequately supported by the results?

Partly

Are sufficient details of methods and analysis provided to allow replication by others?

Partly

Reviewer Expertise:

neonatology

I confirm that I have read this submission and believe that I have an appropriate level of expertise to confirm that it is of an acceptable scientific standard, however I have significant reservations, as outlined above.

# Response

**Published:** 2024-12-20

**Authors:** Sowmini Padmanabh Kamath

**Affiliations:** Kasturba Medical College, Mangalore, India

**1. Mention who took the data; it is better to be the third person who is not involved in the study.**

**Author response: **The duty resident at NICU collected the data.

 We agree that it is better if a third person who is not involved in the study collects the data

**2. Questionnaire validation to be done and mention who prepared**

**Author response: **The questionnaire was prepared by the first four authors after a thorough review of the literature pertaining to the topic. The content was validated by three senior pediatricians working in NICU set-ups. The wordings of the questions with ambiguity were changed as per their suggestions

**3. Sample size justification is needed**

**Author response: **All ambulance personnel of ‘108’ ambulances during the study time were recruited; thus, we did not have a separate sample size calculation

**4. A low sample size is a limitation**

**Author response: **We agree that the low sample size is a limitation. 

**5. Reference for sample size is better to add-**

**Author response: **All ambulance personnel of ‘108’ ambulances during the study time were recruited; thus, we did not have a separate sample size calculation

**6. SPSS country version better to mention-**

**Author response: **New York is written as NY

**7. Cause of attrition is better to mention**

**Author response: **Postintervention, the three APs were excluded since they had been assigned emergency ambulance duties. Thus, for the final analysis, we included 77 APs to assess perceptions and practices

**8. Is it a quasi-experimental study**

**Author response: **It is a single arm intervention (pre-and post) study.

**9. Definitions need citations**

**Author response: **citations included as suggested 

**10. Significance of statistical errors as pre and post-test people are different**

**Author response:** The subject population for AP was the same pre- and post-intervention, except that three APs could not give the post-test questionnaire because of their ambulance duties.

 However, the subject population for outcomes of neonatal transport ought to be different pre- versus postintervention, which could give a possibility of random error/bias.

**11. Ambulance driver’s qualifications may affect the internal study result; Knowing the data better for comparing other studies**

**Author response: **We agree that the qualifications of ambulance personnel would affect the results of the internal study. We have included the educational status of AP in the baseline characteristics part of the results section. However, we did not assess the educational status with their mean perception and practice scores since it was not our study objective; we can include this as a limitation of our study.

**12. Any novelty, better to add**

**Author response: **It is an intervention study that aims to improve the quality of transport of neonates. There is limited literature on pre-versus-post intervention studies on ambulance personnel, specifically ‘108’ ambulances.

# Reviewer report: Reviewer response for version 1

**DOI:** 10.5256/f1000research.164592.r301768

**Published:** 2024-07-22

**Authors:** A Shashidhar Rao

**Affiliations:** 1 Dept of Neonatology, St Johns Medical College Hospital, Bangalore, Karnataka, India

Authors need to be complimented for a social project. Few suggestions

 1. The abstract should be rewritten for better clarity. Include the hypothesis in the background and only the primary objective. Results should include data and not only p values. the data perception component is not presented. (Authors to decide if it is worth presenting).

 2. The introduction can be better organized from general newborn survival/care to the Indian scenario and can be shorter.

 3. The flow of participants is part of the results.

 4. The objectives are a part of introduction and not methods and are better written as a paragraph.

 5. The authors are referred to any other published trial to better organize the methodology section

 7. Results need better clarity and be divided as outcomes of newborns, outcomes of AP etc. baseline characteristics of AP are missing(training, qualification).

 8. There is a significant chance of assessor bias as the assessors are the same as authors

 9. Sample size is not calculated. Could be included even now.

 10. It is unclear if any system changes were made or only training as the changes are expected to last longer.

 11. Embrace needs to be made clear, KMC or mother accompanying is missing, 

 12. QI approach would have been more useful to address this gap.

Is the work clearly and accurately presented and does it cite the current literature?

Yes

If applicable, is the statistical analysis and its interpretation appropriate?

Yes

Are all the source data underlying the results available to ensure full reproducibility?

Yes

Is the study design appropriate and is the work technically sound?

Partly

Are the conclusions drawn adequately supported by the results?

Yes

Are sufficient details of methods and analysis provided to allow replication by others?

Yes

Reviewer Expertise:

Neonatology

I confirm that I have read this submission and believe that I have an appropriate level of expertise to confirm that it is of an acceptable scientific standard, however I have significant reservations, as outlined above.

# Response

**Published:** 2024-12-18

**Authors:** Sowmini Padmanabh Kamath

**Affiliations:** Kasturba Medical College, Mangalore, India

1. **The abstract should be written for better clarity. Include the hypothesis in the background and only the primary objective.**

**Author response**: The abstract is modified for better clarity. The hypothesis and the primary objective have been included in the background

**2. Results should include data and not only p values. The data perception component is not presented (authors to decide if it is worth presenting).**

**Author response**: The data, along with the p values, have been included. We have now presented the data on the perception component.

**3. The introduction can be better organized from general newborn survival/care to the Indian scenario and can be shorter.**

**Author response:** The introduction is organized and modified as suggested.

**4. The flow of participants is part of the results.**

**Author response:** The flow of participants in the consort figure  1 has been included at the beginning of the results section.

**5. The objectives are part of the introduction and not methods and are better written as a paragraph.**

**Author response:** The objectives of the study have been incorporated in the last paragraph of the introduction section.

**6. The authors are referred to any other published trial to better organize the methodology section**

**Author response:** We have reorganized the methodology section as suggested.

  7. **Results need better clarity and can be divided as outcomes of newborns, outcomes of AP etc. Baseline characteristics of AP are missing (training, qualification):**

**Author response:** We have reorganized the results into

- The baseline characteristics of AP and transported neonates –

 The minimum qualification to join an ambulance crew was completing a Secondary School Leaving Certificate(S SLC); few of them had completed the 12th class, and very few had completed their bachelor’s degree. Once they joined, they received 7-day training by the GVK Emergency organization to deal with medical emergency conditions. 

- Outcomes of AP -perceptions and practices.
- Outcomes of transported neonates -described under subheadings primary and secondary outcome measures.
- Ambulance equipment availability and usage
** **

**8. There is a significant chance of assessor bias as the assessors are the same as authors**

**Author response:** As per us, all assessors in the study had adhered to research principles and guidelines. They were unbiased in assessing the perception and practices of AP.

**9. Sample size is not calculated. Could be included even now**

**Author response:** All ambulance personnel of ‘108’ ambulances during the study time were recruited thus we did not have a separate sample size calculation

**10. It is unclear if any system changes were made or only training as the changes are expected to last longer**

**Author response:** There was an improvement in the availability and usage of the following equipment: digital thermometer, glucometer, glucometer strips, neonatal masks, and pulse oximeter post-intervention. The use of hand rubs had improved. System changes have been explained in the results section.

**11. Embrace needs to be made clear, KMC or mother accompanying is missing.**

**Author response:** Embrace infant warmers are portable sleeping bag-like devices that keep low birthweight babies warm in hospital setups and in ambulances that are involved in neonatal transport. It is intended to be utilized in situations when skin-to-skin care is not feasible or when high-tech incubators are not available.

 Adopting Kangaroo mother care will allow skin-to-skin contact between the mother and baby, thereby providing warmth; however, we did not document data on the same since most of the time, the mothers did not accompany the babies that are transported immediately after delivery to our center.   

**12. QI approach would have been more useful to address this gap.**

**Author response:** We agree the QI approach would have been more useful to address this gap and have included this as a limitation in the study
